# Supplementary material for: Membrane Lipid Reshaping Underlies Oxidative Stress Sensing by the Mitochondrial Proteins UCP1 and ANT1
Source: Antioxidants (Basel). 2022 Nov 23;11(12):2314. doi: 10.3390/antiox11122314 (PMC9774536; doi:10.3390/antiox11122314)
Supplement: Supplementary file 1 [file antioxidants-11-02314-s001.zip › antioxidants-1998485-supplementary.pdf]

Supplementary Materials

Membrane lipid reshaping underlies oxidative stress sensing by the mitochondrial proteins UCP1 and ANT1

Olga Jovanović[1], Ksenia Chekashkina[2,3], Sanja Škulj[4], Kristina Žuna[1], Mario Vazdar[5], Pavel V. Bashkirov[2,6], Elena E. Pohl[1,@]

[1]Institute of Physiology, Pathophysiology, and Biophysics, Department of Biomedical Sciences, University of Veterinary Medicine Vienna, 1210 Vienna, Austria; olga.jovanovic@vetmeduni.ac.at (O.J.); kristina.zuna@vetmeduni.ac.at (K.Ž.)
[2]Federal Research and Clinical Center of Physical-Chemical Medicine, 119435 Moscow, Russia; ksenia.chekashkina@gmail.com (K.C.)
[3]A.N. Frumkin Institute of Physical Chemistry and Electrochemistry, 119071 Moscow, Russia;
[4]Department of Chemistry, Faculty of Science, University of Zagreb, 10000 Zagreb, Croatia; sanja.skulj@chem.pmf.hr
[5]Department of Mathematics, University of Chemistry and Technology, 16628 Prague, Czech Republic; mario.vazdar@vscht.cz
[6]Scientific Research Institute of System Biology and Medicine, 117246 Moscow, Russia; pavel.bashkirov@sysbiomed.ru (P.V.B.)
[*]Correspondence: elena.pohl@vetmeduni.ac.at

This PDF file includes:

Supplementary text
Figures S1 to S3

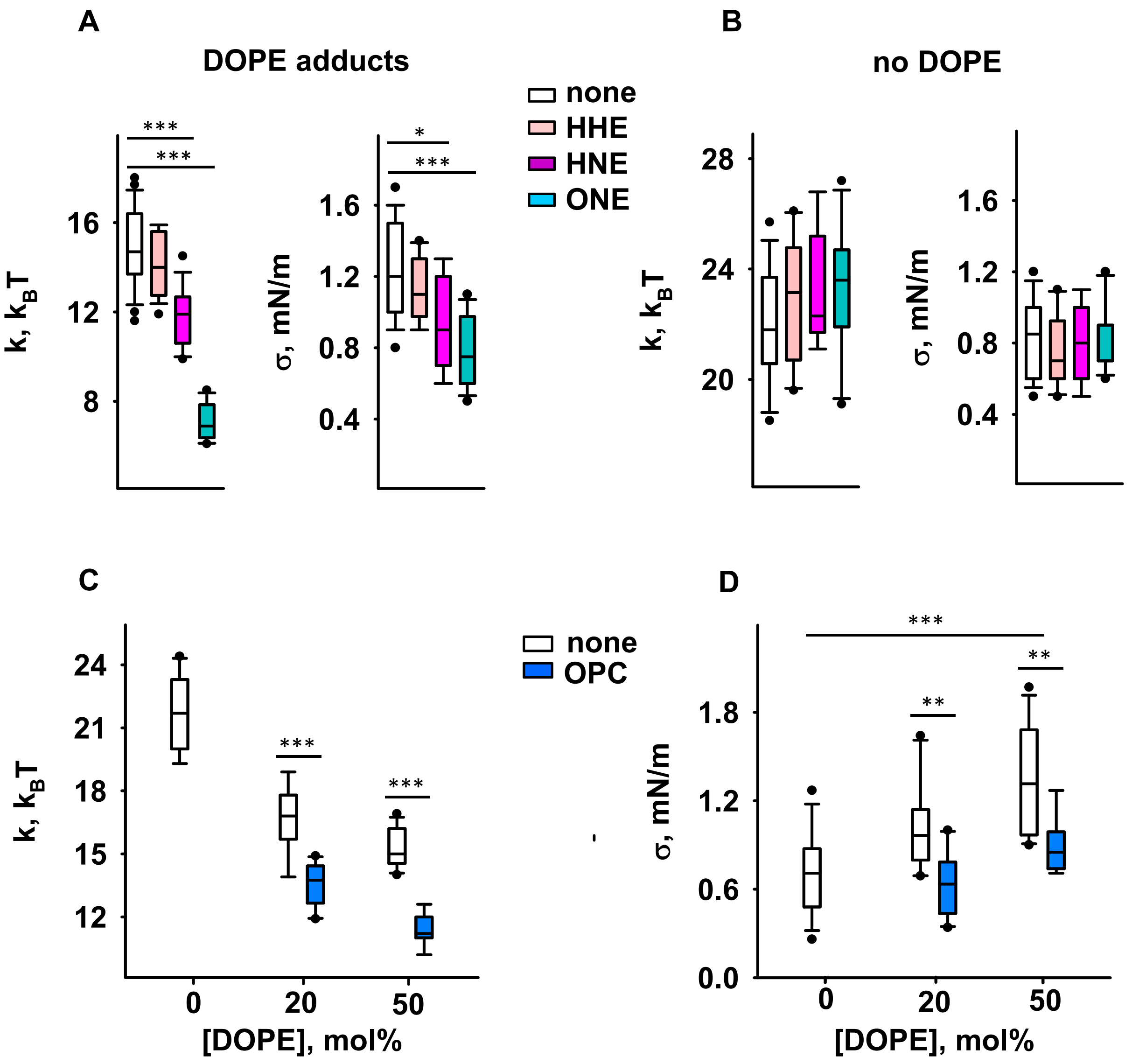

Figure S1. The lipid shape changes the elastic properties of the membrane. A and B. Bending modulus *k* and lateral tension *σ* measured for lipid membranes composed of DOPC:DOPE:CL (45:45:10 mol %, A) and DOPC:CL (90:10 mol%, B). Reactive aldehydes (RAs) were 4-hydroxy-2-hexenal (HHE), 4-hydroxy-2-nonenal (HNE), and 4-oxo-2-nonenal (ONE). RAs concentrations were in the range of (500 – 700) μM. C and D. The impact of different concentration of DOPE on *k* and *σ* of lipid bilayer composed of DOPC (white) or DOPC and OPC (20 mol%, blue). Buffer solution composition as described in Figure 2. Boxes in box-charts indicates mean ± SD from more than ten independent experiments. Whickers represent means ± 95%. * $P<0.05$; ** $P<0.01$; *** $P<0.001$, t-test.

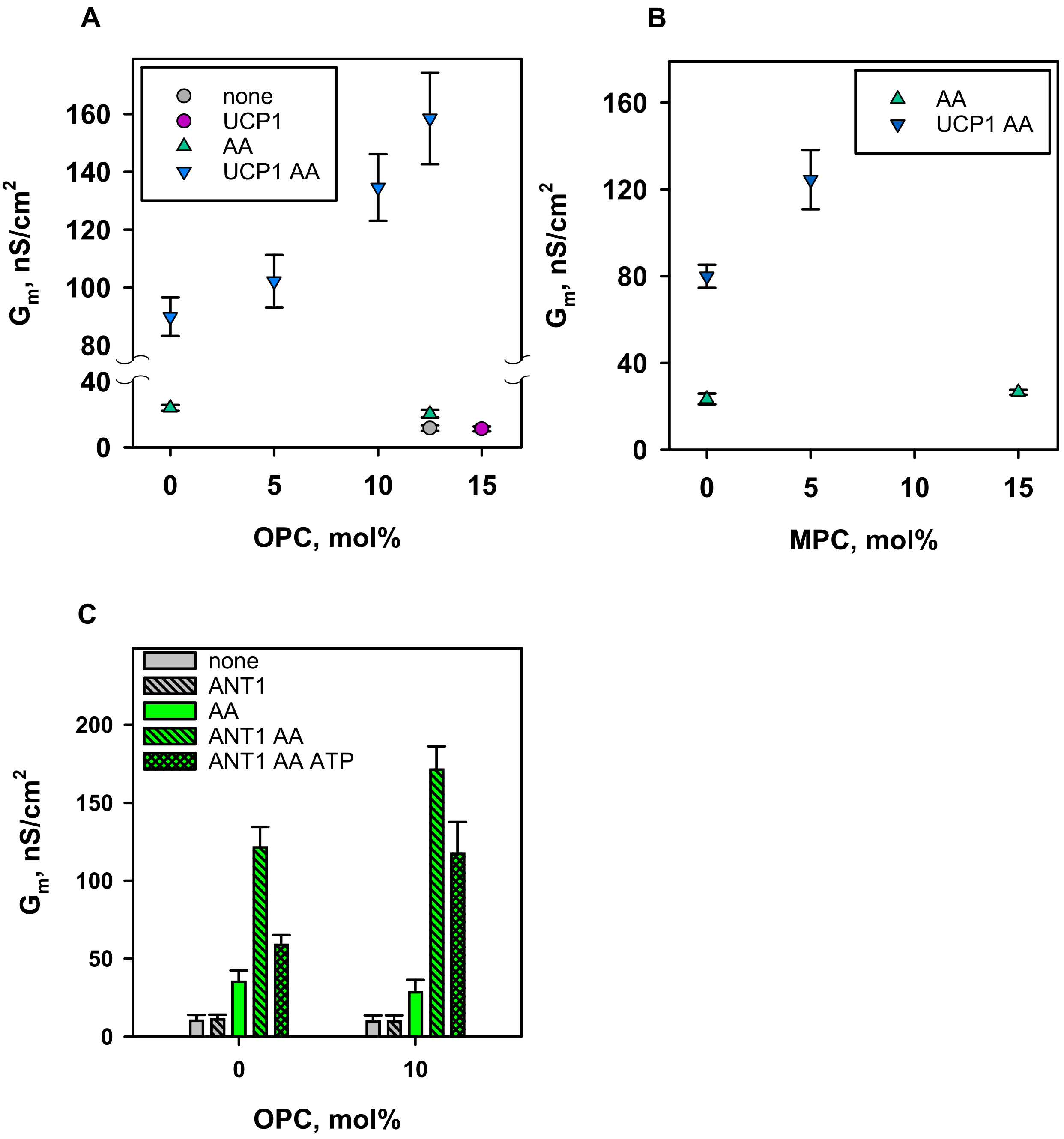

Figure S2. Lipid shape affects UCP1 and ANT1-mediated total membrane conductance (G_m). A. Effect of OPC (18:1) on G_m of membranes reconstituted with UCP1 and/or arachidonic acid (AA), as indicated in the legend. B. Effect of MPC (14:0), on the G_m of membranes reconstituted with UCP1 and/or AA as indicated in the legend. C. Effect of 10 mol% OPC on the G_m value of bilayer membranes reconstituted with ANT1 and/or AA as indicated in the legend. For protein inhibition, 4 mM ATP were added. In the absence of lysolipids the membrane lipid composition was DOPC:DOPE:CL (45:45:10 mol%).

In all experiments, lipid and AA concentrations were 1.5 mg/ml and 15 mol%, respectively. UCP1 concentration was 4-5 µg/mg, and ANT1 concentration was 4 µg/mg. The buffer solution contained 50 mM Na_2SO_4, 10 mM MES, 10 mM TRIS, 0.6 mM EGTA, at pH 7.32 and T = 305 K. Data points represent means and standard deviation from 3-5 independent experiments.

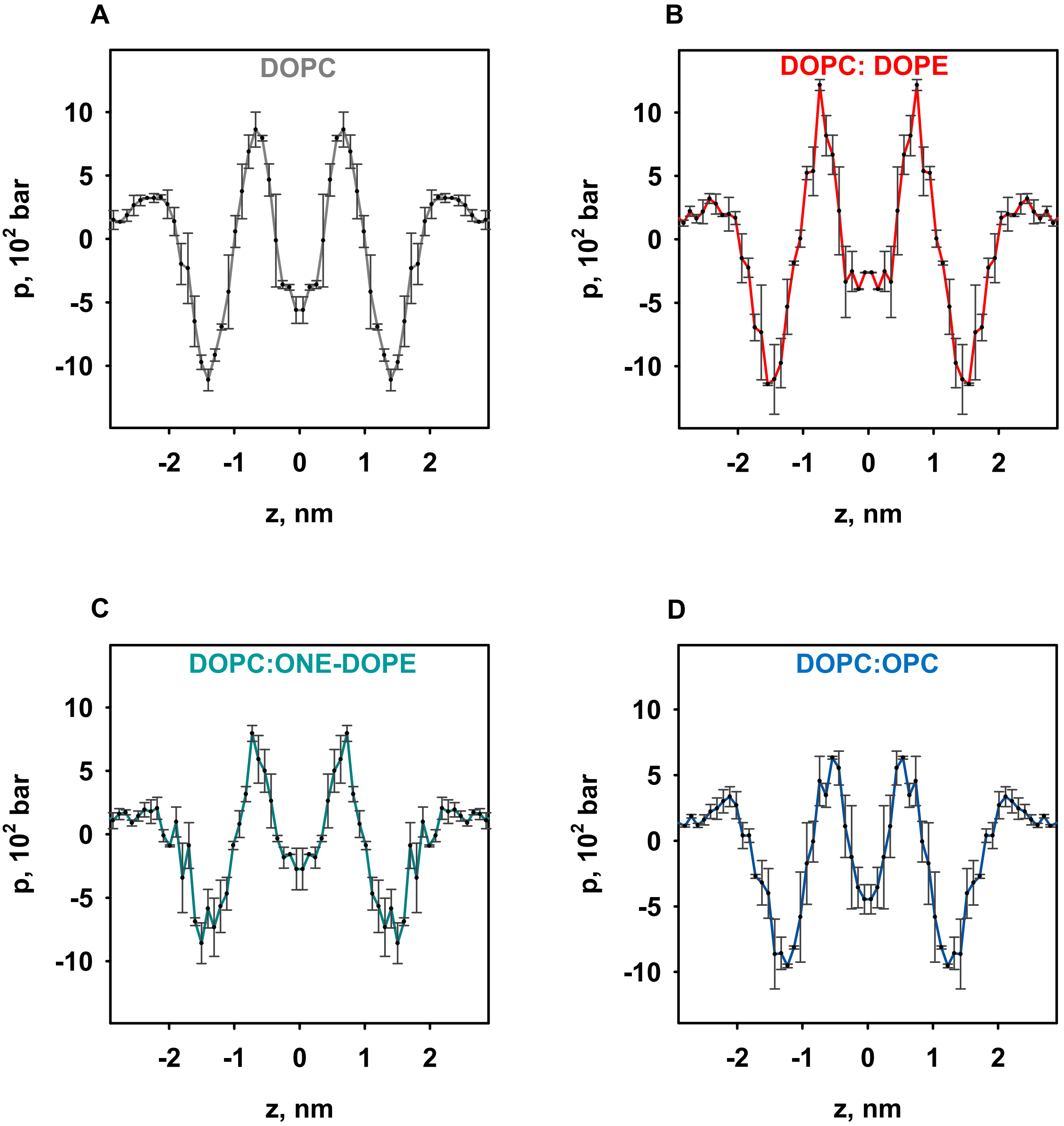

Figure S3. Impact of lipids with pronounced spontaneous curvature on the lateral pressure profile, *p*. The lipid ratio was 50:50 mol% in two-component bilayers. The error bars for the pressure profiles of lipids were calculated as the difference between the symmetrized and unsymmetrized pressure profiles in the different leaflets. Smoothed data were obtained from the average of two points.
